# Factors affecting free vancomycin concentration and target attainment of free area under the concentration-time curve

**DOI:** 10.1186/s40780-025-00419-4

**Published:** 2025-02-18

**Authors:** Toshiharu Urakami, Yusuke Oka, Takashi Matono, Yosuke Aoki

**Affiliations:** 1 Division of Infectious Disease and Hospital Epidemiology, Saga University Hospital, 5-1-1, Nabeshima, Saga, 849- 8501 Japan; 2 Department of International Medicine, Faculty of Medicine, Saga University, Saga, Japan

**Keywords:** Vancomycin, Protein binding rate, Therapeutic drug monitoring, Area under the concentration-time curve, Free area under the concentration-time curve, Hyper-proteinemia, Hypo-proteinemia

## Abstract

**Background:**

It has been reported that the protein binding rate of vancomycin (VCM) varies among individual patients. So, the authors investigated relevant factors that may affect free VCM concentration and target attainment of free area under the concentration-time curve (fAUC).

**Methods:**

Thirty-nine patients were included. Multiple regression analysis was performed to determine the valuable factors in the free VCM concentration, and the target attainment of area under the concentration-time curve (AUC) 400–600 mg・h/L and fAUC200-300 mg・h/L was calculated.

**Results:**

We found total protein was significant covariate for free VCM. Among 18 patients who were investigated for AUC and fAUC estimation, 9 patients (50.0%) and 12 patients (66.7%) reached AUC > 600 mg・h/L, and fAUC > 300 mg・h/L (*p* = 0.310), respectively.

**Conclusions:**

Total protein is a significant predictor for free VCM estimation. And the fAUC-guided TDM for VCM TDM may contribute to more strict dosing than the AUC-guided TDM in hyper- or hypo-proteinemic population.

**Trial registration:**

Retrospectively registered.

## Background

Vancomycin (VCM) is a glycopeptides that has antibacterial activity against penicillin-resistant Gram-positive cocci such as methicillin-resistant *Staphylococcus aureus* (MRSA) and *Enterococcus faecium* [1]. Usefulness of the pharmacokinetic/pharmacodynamic (PK/PD) for predicting clinical efficacy and safety has been well established. Although, PK/PD indices practically focuses on the total (protein-bound and free) drug concentration, only the free concentration has antibacterial activity at infection site, warranting the need to measure free-drug concentration. Therapeutic efficacy of quinolones and beta-lactams correlates with PK/PD indices based on free concentration [2, 3]. For VCM, area under the concentration-time curve (AUC)/ minimum inhibitory concentration (MIC) ratios correlated with slowly bactericidal effect. Moreover, PK/PD indices have recently been applied as therapeutic ranges for therapeutic drug monitoring (TDM) of VCM. Because the MIC of VCM is generally 1 µg/mL judging from the MIC distribution for MRSA, the target AUC is equal to the target AUC/MIC. The TDM for VCM is recommended for AUC/MIC 400–600 mg・h/L so as to improve efficacy and reduce nephrotoxicity [4]. In addition, the free area under the concentration-time curve (fAUC)/MIC has been recommended as a more desirable PK/PD target for VCM [5]. Considering conservative protein-binding rate of 50%, fAUC/MIC of 200–300 mg・h/L has been targeted. However, there are few studies on fAUC guided TDM for VCM therapy. The aim of this study is to identify relevant factors influencing free VCM concentration and evaluate target attainment of fAUC.

## Methods

### Study design and the patients

This single-center, prospective and observational study enrolled hospitalized patients over 18 years of age whose total and free VCM concentration were measured, to whom VCM was administered intravenously in the Saga University Hospital. The study period was from January 2021 to December 2023 (36 months).

### VCM determination

Blood samples to determine total and free VCM concentration were drawn during daily routine TDM at timings judged appropriate by medical doctors. Blood samples were then centrifuged at 5,000 × g for 10 min. The supernatant was separated immediately and measured for the total VCM concentrations using particle-enhanced turbidimetric inhibition immunoassay(Dimension^®^ XpandⓇ, SIEMENS). This method is comparable with high-performance liquid chromatography in vancomycin measurements [6]. So, after the free fraction was collected by a validated ultra-filtration method with Centrifree^®^ centrifugal filter unit (molecular weight cut-off, 30 kDa; Merck Millipore) at 2,000×g (37 ℃) for 30 min [7, 8]., free concentration was measured in the same way as total concentration. Protein binding rate (%) was measured as follows: [(total concentration - free concentration)/total concentration] × 100. In order to estimate AUC, blood sampling was drawn on or after the 3rd day of VCM administration at steady-state. Those patients whose timing of sample collection were not likely at trough level or whose dosing interval were over 12 h were excluded. Bayesian software [Practical AUC guided TDM for vancomycin (PAT) ver.3.0a] constructed by the Japanese Society of Chemotherapy was used to calculate AUC with trough level only [9]. The fAUC was calculated as follows: [(100-protein binding rate)/100] × AUC.

### Data collection

Baseline data was extracted for age, sex, total protein, serum creatinine, location (ICU or non-ICU), the presence or the absence of malignancy, and VCM dosing regimen from patients’ medical record. Creatinine clearance (CLcr) was estimated by using the Cockcroft-Gault formula. In the case that biochemical data was not measured on the same day of sample collection, the closest value within 3 days of sample collection was included. Target attainment based on AUC 400–600 mg・h/L and fAUC 200–300 mg・h/L were calculated.

### Statistical analysis

Statistical analysis was performed with JMP Pro 16.2.0 for Windows (SAS Institute Inc., USA). Data was recorded as median and interquartile range (IQR). A p value of < 0.05 was considered statistically significant difference. Linear least-squares regression was performed for between total and free concentrations. Multivariate linear regression was derived to determine relevant factors for estimating free concentration. The number of explanatory variables, which may be generally based on one-tenth of the estimated sample size, was determined as four. Total vancomycin, total protein, ICU admission, and being a hematology patient were selected as explanatory variables with reference to previous research on free VCM [8, 10, 11]. Target attainment based on AUC and fAUC was assessed by scatterplot and chi-squared test.

## Results

**Fig. 1:**
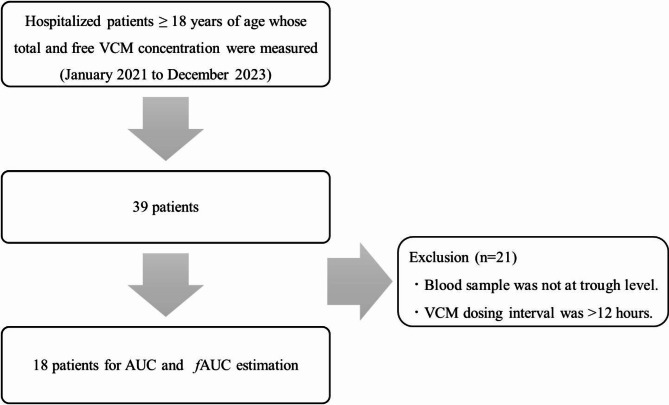
Flowchart of patient inclusion. Abbreviations: VCM, vancomycin; AUC, area under the concentration-time curve; fAUC, free area under the concentration-time curve

**Fig. 2:**
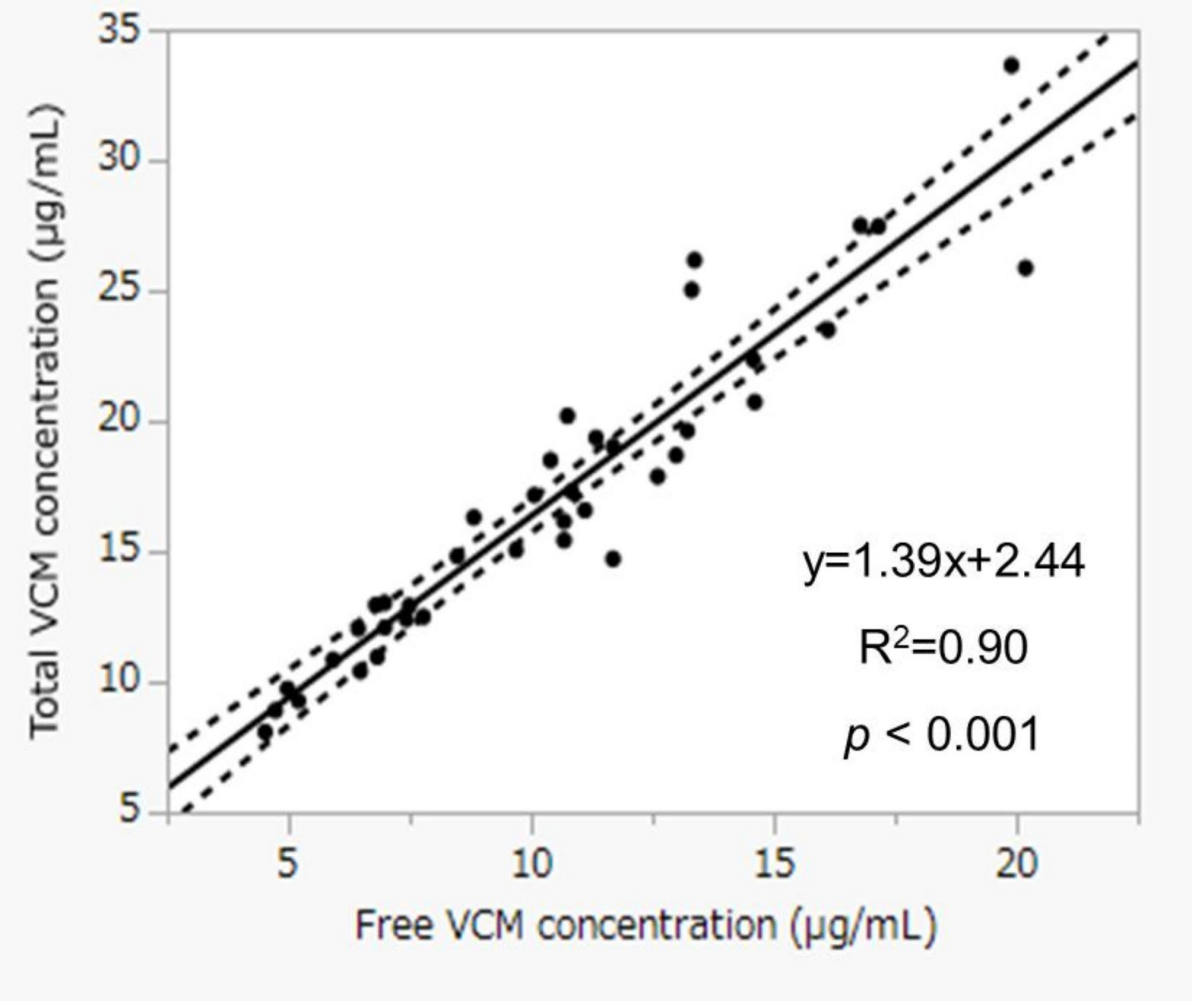
Correlation between free VCM concentration and total VCM concentration (*n* = 39). The dotted lines indicate the 95% confidence interval. Abbreviations: VCM, vancomycin

**Fig. 3:**
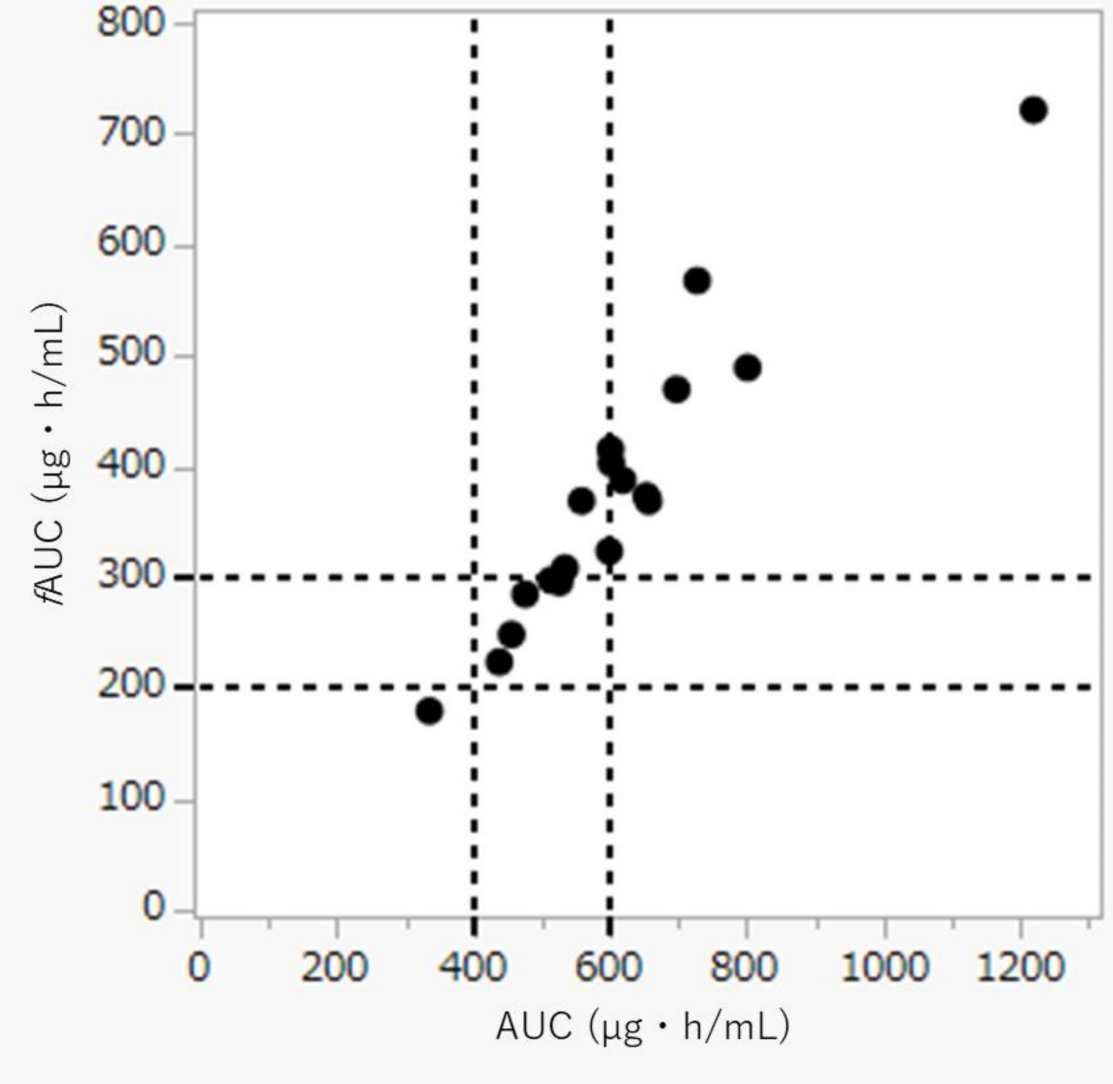
Target attainment based on AUC and fAUC (*n* = 18). The broken lines indicate therapeutic range, specifically AUC 400–600 and fAUC 200–300. The area between broken line is therapeutic range for AUC and fAUC of VCM. Abbreviations: VCM, vancomycin; AUC, area under the concentration-time curve; fAUC, free area under the concentration-time curve

The number of patients employed for the study was 39; 18 patients were calculated both AUC and fAUC among them (Fig. 1). Table 1 shows the patient characteristics at baseline. Both total and free concentrations were measured in each patient. The median protein binding rate (IQR) was 40.1% (34.3–44.8%). Judging from linear regression of total and free concentrations, coefficient of determination (R^2^) was 0.90 (*p* < 0.001), resulting in statistical significance (Fig. 2). Result of multivariate linear regression, total VCM concentrations [β = 0.611, standard error of the mean (SEM) = 0.029, *p* < 0.001] and total protein (β = -1.083, SEM = 0.256, *p* < 0.001) were found to be significant covariates for free fraction (Table 2). The prediction model for free VCM concentration is as follows: free VCM [µg/mL] = 5.3 + 0.6 × total VCM [µg/mL] − 1.1×total protein [g/dL]. The AUC was estimated in 18 patients (18 samples) using PAT. The median AUC, and fAUC was 599.9 mg・h/L (IQR = 516.6 mg・h/L − 655.6 mg・h/L) and 369.1 mg・h/L (IQR = 296.2 mg・h/L − 412.7 mg・h/L), respectively. Nine patients (50.0%) reached AUC > 600 mg・h/L, and 12 patients (66.7%) reached fAUC > 300 mg・h/L (Fig. 3). No significant differences were observed in probability of target attainment significantly (*p* = 0.310).

**Table 1:** Patient characteristics at baseline

Factors	Total (*n* = 39)	Cases with AUC and fAUC calculated (*n* = 18)
Sex, *n* (%)		
Male	24 (61.5)	11 (61.1)
Age, years		
Median (IQR)	68 (62.0–78.0)	67 (62.0–74.3)
Age group, n (%)		
≥70 years	15 (38.5)	5 (27.8)
Serum creatinine, mg/dL		
Median (IQR)	0.9 (0.6–1.8)	0.6 (0.5–0.7)
Creatinine clearance*, n (%)		
< 30 mL/min	12 (30.8)	0 (0.0)
Total protein, mg/dL		
Median (IQR)	5.6 (4.7–6.3)	6.0 (5.5–6.4)
Underlying medical conditions, n (%)		
ICU admission Malignancy	6 (15.4) 12 (30.8)	1 (5.6) 4 (22.2)

* Creatinine clearance was estimated by using the Cockcroft-Gault formula. AUC, area under the concentration-time curve; fAUC, free area under the concentration-time curve; ICU, intensive care unit; IQR, interquartile range

**Table 2:** Significance of influence of clinical and pharmacological factors on free Vancomycin concentration

Variable	β	SEM	*P* value
Total Vancomycin	0.611	0.029	< 0.001
Total protein	-1.083	0.256	< 0.001
ICU admission (yes = 0, no = 1)	0.023	0.246	0.926
Malignancy (yes = 0, no = 1)	0.179	0.200	0.379

β, beta coefficient, SEM, standard error of the mean (multivariate linear regression). ICU, intensive care unit

## Discussion

In this study, we demonstrated that protein binding rate of VCM is 40%, although being highly variable. These data provide a basis for reconsidering the importance of free concentration in VCM therapy. We have shown that free VCM concentration was influenced by total serum protein concentration. Therefore, free VCM concentration may be a better indicator of clinical outcome than total VCM concentration in patients with hyper- or hypoproteinemia. In Fig. 3, we compared target attainment AUC and fAUC, and no significant differences were observed. On the other hand, our study suggested it was possible that the protein binding rate of VCM was lower (40%) than as has been reported (50%) [12]. Given that Butterfield et al.. also reported a similar result to ours [10], future studies should be based on new protein binding rate (40%) instead of conservative protein binding rate (50%) for vancomycin. Despite free concentration is supposed to be better indicator than total as PK/PD indices, the Japanese guideline recommended an AUC target of 400–600 mg・h/L based on total VCM concentration presuming MIC being 1 µg/mL [5]. It is not ideal that the AUC-guided TDM is generally conducted for VCM. The fAUC-guided TDM should rather be more appropriate for VCM therapy. However, fAUC target has not been examined sufficiently. Judging from protein biding rate of 40% in our study, the target fAUC may be 240–360 mg・h/L for the future. Likewise, Leroux, et al. reported that AUC/MIC of 400 mg・h/L corresponds to fAUC/MIC of 240 mg・h/L, assuming median protein binding rate of 40% in adult [13]. In addition, fAUC is supposed to be the most appropriate PK/PD indices. Rybak has stated 24-h bacterial count of methicillin-sensitive *S. aureus* correlates with AUC in neutropenic mouse thigh infection model [14]. However, one should note that the correlation between AUC and antimicrobial effect was evaluated by semi-quantitative interpretation, and the target value of fAUC was not mentioned in this review. Further animal study is necessary to investigate the target fAUC of VCM.

In this study the measurement of free VCM concentration raises two important issues. The first one is high variability in protein binding rate. Furthermore, protein binding rate of VCM varied in this study. Moise-Broder also reported that the protein binding of this drug ranged from 29 to 71% [15]. Berthoin, et al. reported that there was a significant variation in protein binding rate (12–100%) [16]. These variabilities indicate that free VCM concentration cannot be predicted precisely. Therefore, although vancomycin is binding-insensitive, we should pay attention to free concentration to practice more precise TDM. The second is the influence of hypoproteinemia. We found serum total protein was a significant covariate for free VCM concentration in multivariate linear regression since VCM is predominantly bound both by albumin and immunoglobulin A [17]. However, albumin and total protein are potentially confounding, and therefore we did not select albumin as an explanatory variable in multivariate linear regression [10, 17, 18]. From these findings, we should adopt free VCM target approach, particularly in hyper- or hypo-proteinemic population.

Since few studies have measured the fAUC of VCM, we calculated fAUC based on the protein binding rate and the AUC by the Bayesian method. We estimated AUC by only the trough level. Since K. Oda, et al. reported that AUC estimation solely based on the trough level produced strong bias in patients receiving VCM every 24 h [9], we targeted the patients receiving VCM every 12 h and less for AUC estimation. The Japanese guideline recommended target AUC is 400–600 mg・h/L. Given that vancomycin protein binding rate is 50%, the therapeutic range of fAUC is broadly 200–300 mg・h/L [19]. In our study, 9 patients (50.0%) had an AUC > 600 mg・h/L and 12 (66.7%) had an fAUC > 300 mg・h/L. No significant differences were observed in the probability of target attainment. Therefore, we should perform AUC-guided TDM in principle. On the other hand, the above result suggests that TDM based on fAUC may strictly reduce the VCM dosage in a selected population. Namely, the fAUC-guided TDM for VCM has the potential to reduce side effects such as acute kidney injury in hypoproteinemic population.

Our study has limitations. First, this is a single-center noninterventional enrolling limited number of patients. Second, we had not performed correlation between free vancomycin concentration as determined by high-performance liquid chromatography and by the particle-enhanced turbidimetric inhibition immunoassay. Third, we did not investigate the impact of liver disease, concomitant medications and serum immunoglobulin concentration on free vancomycin. Because this is a retrospective study, we were unable to accurately calculate the Child-Pugh Score. And the influence of drug-drug interactions on protein binding of vancomycin is complex and difficult to assess comprehensively. Because immunoglobulin is a part of total protein in serum, we used only serum total protein for free VCM estimation. Lastly, we couldn’t compare the target attainment rates of AUC and fAUC dividing into hyperproteinemia and hypoproteinemia groups because of small sample size, so further research is needed.

## Conclusions

We conclude that protein binding rate of VCM was 40% with variability. No significant differences were observed between AUC and fAUC in probability of target attainment. Therefore, we should perform AUC-guided TDM in principle. Total protein is a significant predictor for free VCM estimation, therefor fAUC-based TDM may contribute to strict-dosing regimen, especially in hypoproteinemic population.

## Data Availability

No datasets were generated or analysed during the current study.

## Abbreviations

AUC: Area under the concentration time curve
CLcr: Creatinine clearance
fAUC: Free area under the concentration-time curve
IQR: Interquartile range
MIC: Minimum inhibitory concentration
MRSA: Methicillin-resistant *staphylococcus aureus*
PK/PD: Pharmacokinetic/pharmacodynamic
SEM: Standard error of the mean
TDM: Therapeutic drug monitoring
VCM: Vancomycin
